# Case report: Intracranial epidermoid cyst in a cat

**DOI:** 10.3389/fvets.2024.1426421

**Published:** 2024-09-23

**Authors:** Masashi Terao, Takashi Uemura, Hiroki Hasegawa, China Ashida, Ikuya Ehara, Tsuyoshi Ozawa, Hiroaki Kamishina

**Affiliations:** ^1^KyotoAR Animal Referral Medical Center, Kumiyama, Japan; ^2^St. Luke's Animal Medical Center, Toyonaka, Japan

**Keywords:** brain, cholesteatoma, craniectomy, epidermoid cyst, feline, fourth ventricle, intracranial cyst, MRI

## Abstract

A 9-year-old American Shorthair, castrated male, weighing 4.3 kg was presented to our hospital because of intermittent ataxia and tetraparesis for 6 weeks. On presentation, the cat was in a stupor and on recumbency, and had vertical nystagmus in both eyes. These clinical signs suggested a brainstem disorder. MRI showed a mass lesion in the caudal aspect of the fourth ventricle with hyperintensity on T2-weighted and FLAIR imaging, low-intensity on T1-weighted imaging, and enhanced margins on post-contrast T1-weighted imaging. The mass compressed the fourth ventricle, causing obstructive hydrocephalus. A second cystic lesion was found rostral to the cerebellum. After MRI, the cat experienced respiratory difficulties and the mass was removed by emergency craniectomy. Although the mass including the cyst wall was successfully removed, the cat was euthanized because spontaneous breathing did not return. The mass was histopathologically diagnosed as epidermoid cyst. A biopsy to the rostral cystic lesion had not been performed and therefore the etiology of this lesion remained unclear. This is the first case of feline intracranial epidermoid cyst in which MRI and surgical excision were performed. MRI findings were similar to those in humans and dogs, suggesting that imaging studies are useful in cats for the diagnosis of intercranial epidermoid cyst.

## Introduction

Epidermoid cysts are benign cystic lesions in which epidermal cells ectopically migrate during development (1–3). They form cysts lined by stratified squamous epithelium, inside which exfoliated keratinized material accumulates and gradually enlarges. It is also called a cholesteatoma because of its macroscopic features (3, 4).

Intracranial epidermoid cysts often develop in the cerebellopontine angle and fourth ventricle. As they gradually enlarge, they compress the cerebellum and medulla, causing neurological signs, such as central vestibular disorders (2, 4). These lesions have been reported in humans, horses, dogs, and rats (2, 5–7), and only one case has been reported in a cat (8). In this report, no imaging or surgery was performed and the patient was euthanized and the diagnosis was made at necropsy.

In the present case, we had an opportunity to surgically remove an epidermoid cyst that developed caudal to the fourth ventricle of a cat. To the best of our knowledge, there have been no previous reports of surgical removal or magnetic resonance imaging (MRI) of intracranial epidermoid cysts in cats.

## Case presentation

The case was a 9-year-old American Shorthair, neutered male, weighing 4.3 kg. For 6 weeks the cat had displayed intermittent tetraparesis and proprioceptive ataxia. The cat had no other clinical signs, with normal activity and appetite. No abnormalities were found in physical examination, complete blood count (CBC), blood biochemical test, and cardiac ultrasonography at the primary care clinic. No treatment was instituted at this time. However, abnormal gait continued intermittently and the cat fell down the stairs once. A few days after falling, the cat visited the emergency hospital following collapse and respiratory distress. Chest and abdominal radiography revealed no abnormalities. The patient was referred to the neurology department of our hospital.

At presentation, the cat was in a stupor state with a recumbent posture. It showed tachycardia (200 bpm) and hyperthermia (39.3°C). The cat was non-ambulatory tetraparetic, and showed absent postural reactions, mildly increased spinal reflexes, and decreased superficial pain perception in all limbs. Vertical nystagmus in both eyes was noted, which was exacerbated by placing the cat in dorsal-recumbency. The resting pupillary diameter of both eyes was mid-range, but both direct and indirect pupillary light reflexes were decreased. CBC and blood biochemical tests were unremarkable. Neurological examination was consistent with a brainstem disorder, and MRI of the brain was indicated.

**Figure 1:**
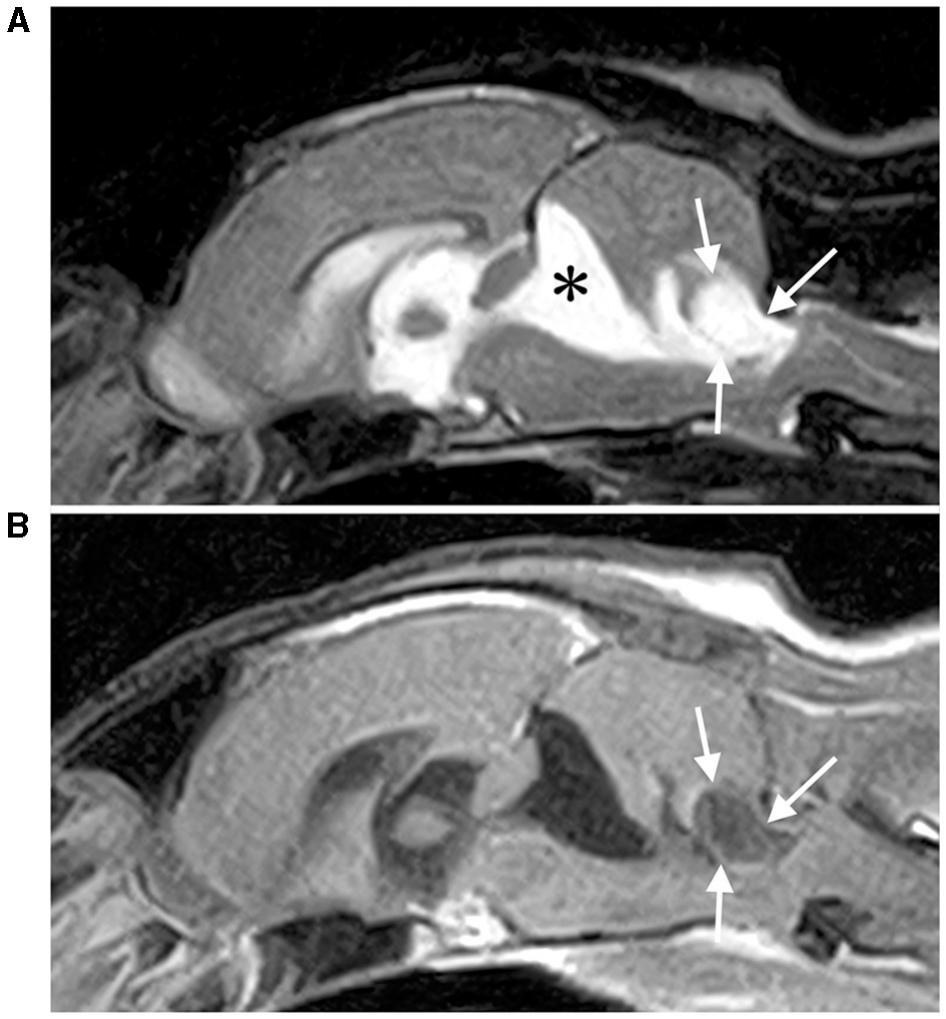
T2-weighted **(A)** and contrast-enhanced T1-weighted **(B)** sagittal MRI of the brain. An extra-axial cystic mass (white arrows) is observed caudal to the fourth ventricle and is compressing the fourth ventricle and medulla severely. A dilation of a second cystic lesion was also observed rostral to the cerebellum (*), which was considered an arachnoid diverticulum.

**Figure 2:**
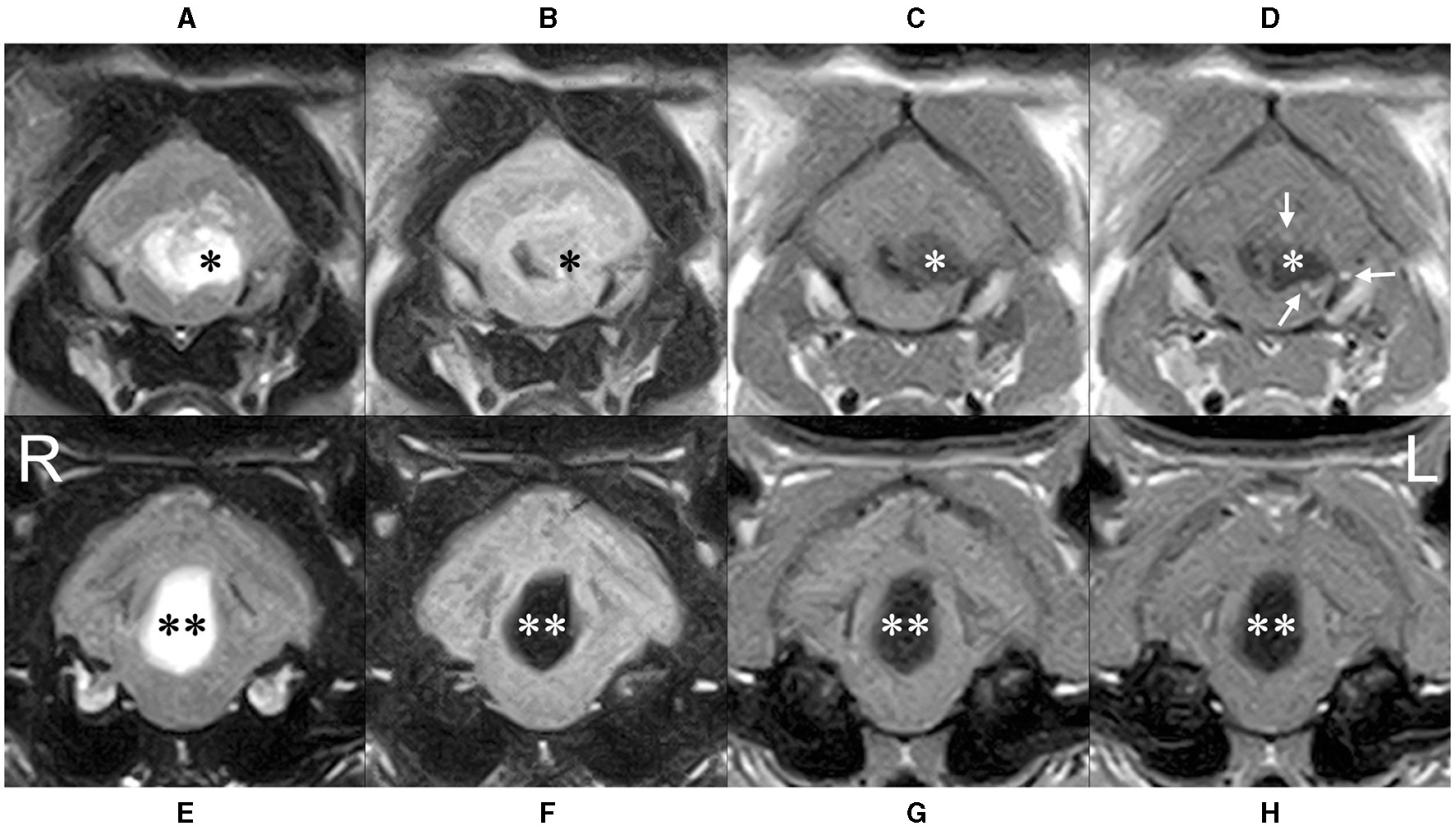
Transverse MRI at caudal **(A–D)** and rostral **(E–H)** level of the cerebellum. The cyst (*) severely compressed the fourth ventricle to the right, the medulla ventrally, and the cerebellum dorsally. High intensity on T2-weighted **(A)** and FLAIR **(B)** imaging, low intensity on T1-weighted imaging **(C)** (higher than cerebrospinal fluid) was observed, and contrast-enhanced T1-weighted imaging **(D)** showed a ring-shaped enhancement of the limbus (white arrows). The rostral cystic lesion (**) showed high intensity on T2-wighted imaging **(E)**, lower intensity than that of cerebrospinal fluid on FLAIR imaging **(F)** and low intensity on T1-wighted imaging **(G)** without any contrast enhancement **(H)**.

The patient was premedicated with midazolam (Dormicum, Maruishi Pharmaceutical Co., Osaka, Japan; 0.2 mg/kg IV) and anesthetized with propofol (PropoFlo28, Zoetis Japan, Tokyo, Japan) injection to effect. After endotracheal intubation, general anesthesia was maintained with 2% sevoflurane (SEVOFLO, Maruishi Pharmaceutical Co., Osaka, Japan). MRI was performed using 0.3T AIRIS Vento (Hitachi, Tokyo, Japan) with sagittal, transverse and dorsal planes on T2-weighted (TR/TE = 4,000/100) imaging; transverse planes on T1-weighted (TR/TE = 380/15) imaging; and fluid-attenuated inversion recovery (FLAIR; TR/TE = 9,000/100) imaging. Sagittal, transverse and dorsal planes of T1-weighted imaging after IV injection of gadolinium (OMNISCAN 32%, GE HealthCare Pharma Co., Tokyo, Japan; 64 mg/kg IV) were also performed. MRI revealed an extra-axial mass lesion at the dorsal medulla and the caudal fourth ventricle, compressing the medulla ventrally and the cerebellum dorsally (Figure 1). The mass was high-intensity on T2-weighted and FLAIR imaging, low-intensity on T1-weighted imaging (higher than the signal of cerebrospinal fluid) and was enhanced in the margins on post-contrast T1-weighted imaging (Figure 2). The third ventricle, lateral ventricles, and olfactory ventricle were enlarged, and the sulci were obscured throughout. Based on these findings, we suspected that the patient had obstructive hydrocephalus secondary to a mass lesion caudal to the fourth ventricle. Epidermoid cysts, arachnoid diverticula, abscesses and neoplasms (e.g., meningioma, lymphoma and ependymoma) were differential diagnoses for the mass (8–11). A histopathological examination was considered necessary for diagnosis. A second cystic lesion was found between the tectum and the cerebellum, dorsal to the quadrigeminal cistern. This lesion showed the same signal pattern as cerebrospinal fluid: high intensity on T2-weighted imaging, low intensity on T1-weighted imaging and FLAIR imaging and no contrast enhancement. This concurrent lesion was suspected to be an arachnoid diverticulum, but the clinical significance of the lesion was unclear.

Although recovery was delayed after MRI, the patient was extubated temporarily after administration of concentrated glycerin (GLYCEOL, TAIYO Pharma Co., Tokyo, Japan; 1 g/kg slow IV), prednisolone (Prednisolone, Kyoritsu Seiyaku Co., Tokyo, Japan; 1 mg/kg SC), furosemide (Flosemide injection, Nichi-Iko Pharmaceutical Co., Toyama, Japan; 1 mg/kg IV). However, we reintubated when the patient stopped breathing and spontaneous respiration did not resume after which emergency craniectomy was performed.

**Figure 3:**
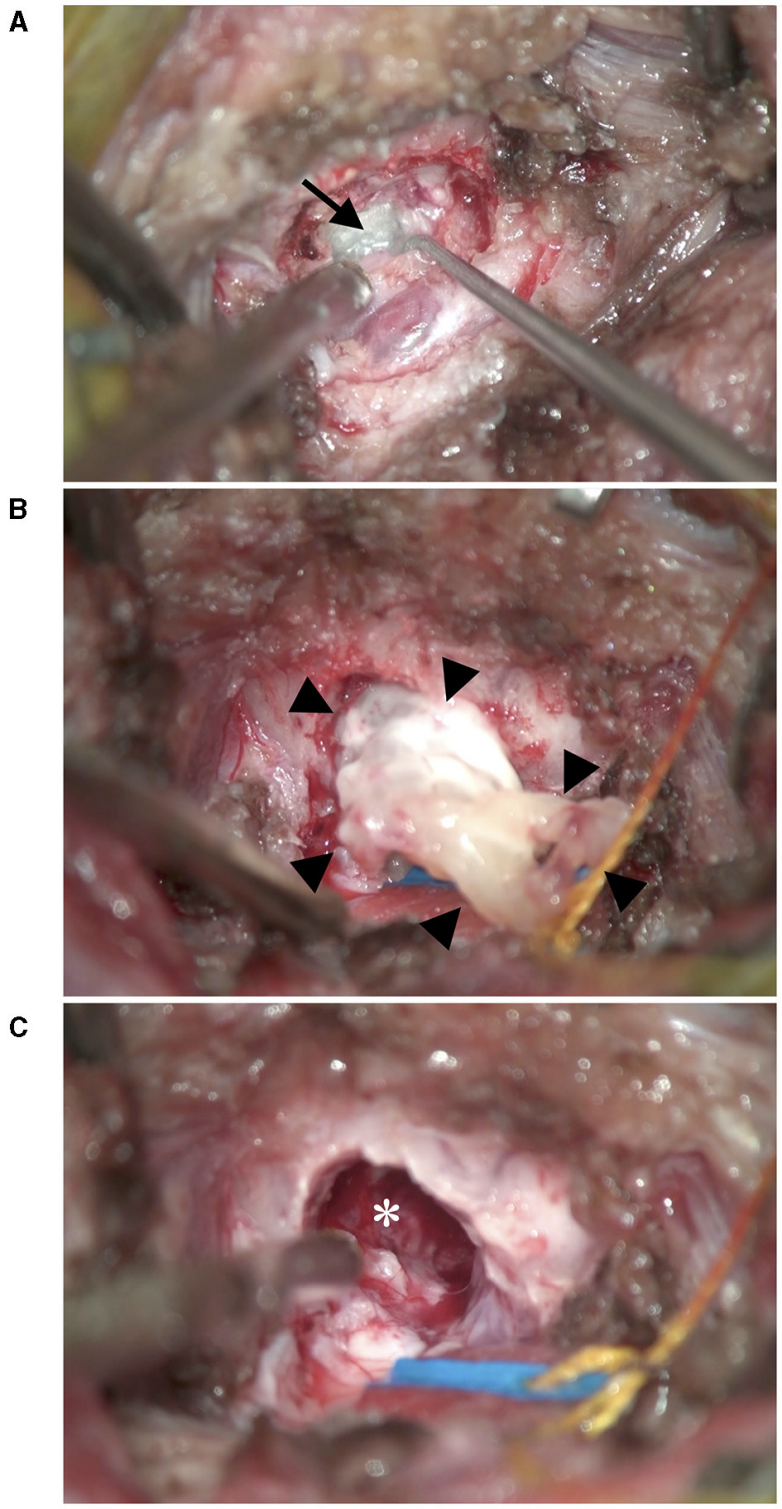
Intraoperative photographs. **(A)** After incision of the dura, a glossy white mass (black arrow) was revealed ventral to the cerebellum. **(B)** After removal of the contents of the mass, the cyst wall (black arrow heads) was excised. **(C)** After removal of the mass, the brain parenchyma did not relocate and remained depressed (*).

A midline incision was made for an occipital approach to the dorsal medulla. After dural incision, a slightly glossy white mass was observed on the ventral side of the caudal border of the cerebellum (Figure 3). The mass was firmer than the brain parenchyma and had a cystic structure. Intraoperative imprint cytology of the contents of the cyst with Diff-Quick staining (SYSMEX Co., Kobe, Japan) revealed denucleated keratinocytes. No bacterial pathogens were observed. After internal decompression by excising the contents of the cyst, the cyst wall was excised. The brain parenchyma did not relocate and remained depressed after mass removal. We covered the craniectomy defect with a free flap of temporalis fascia and applied fibrin glue (Beriplast P Combi-Set Tissue adhesion, CSL Behring K.K., Tokyo, Japan). The wound was closed by a standard method. The excised lesions were fixed in 10% formalin solution. No cultural examination was performed.

Fentanyl (DAIICHI SANKYO Co., Tokyo, Japan; 3 μg/kg/h constant rate infusion) for analgesia and cefazolin (Sefazolin sodium, KOA ISEI Co., Yamagata, Japan; 20 mg/kg IV) as antibiotic were administered during surgery with crystalloid fluid infusion. Post-operatively, the patient did not resume spontaneous respiration and was euthanized after 22 h of ventilator management. Necropsy was not performed.

**Figure 4:**
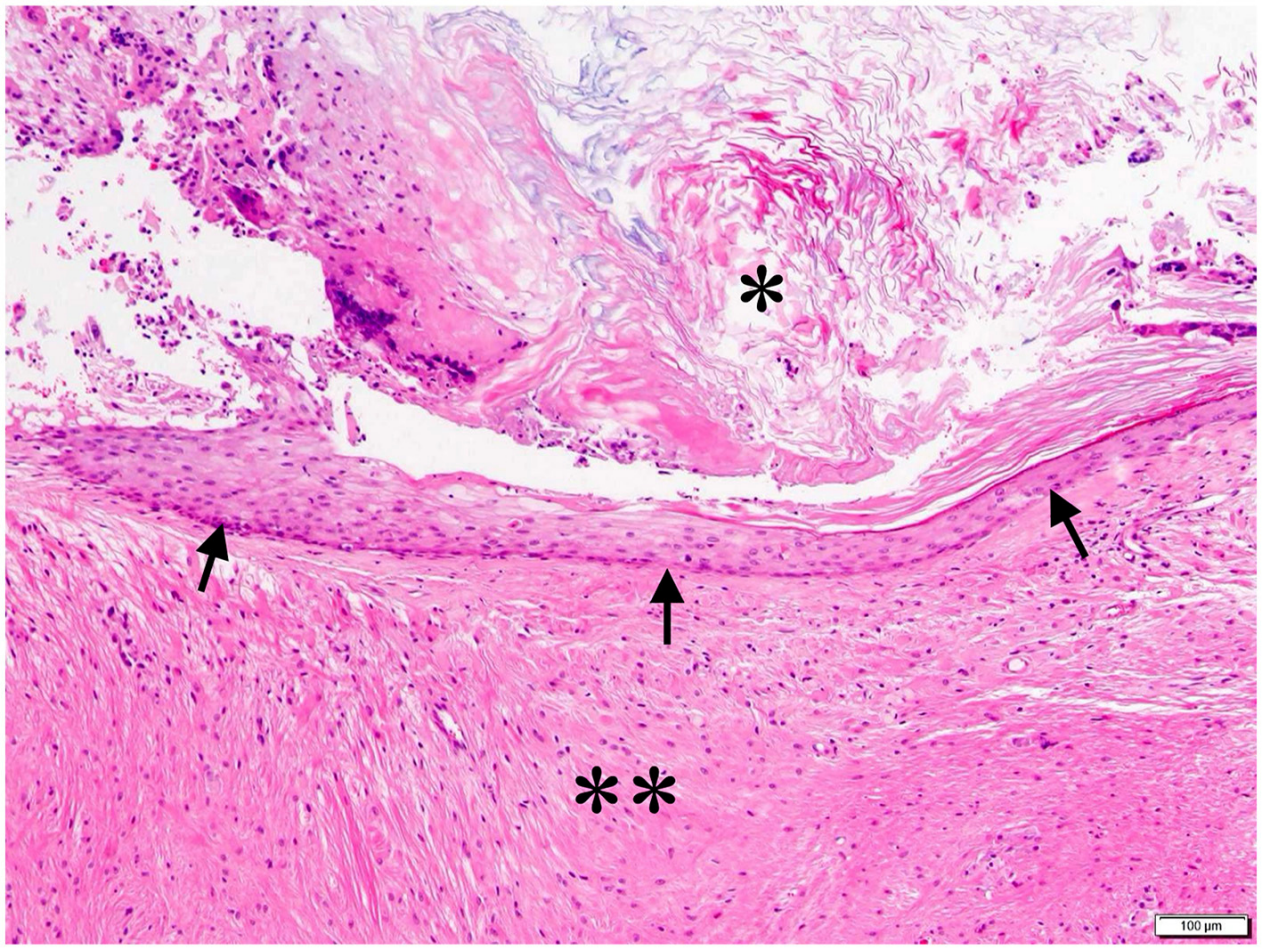
Histopathological image of the cyst. The keratinized stratified squamous epithelium formed the cyst wall (black arrow), and keratin accumulated in the cyst cavity (*). Multinucleated giant cells and macrophage infiltration and fibrous gliosis were observed in the surrounding cerebellar white matter with multilayered keratinization (**). HE stain; bar = 100 μm.

Histopathological examination of the submitted tissue was consistent with intracranial epidermoid cyst (Figure 4). The cyst wall was composed of inwardly keratinized stratified squamous epithelium, and the cyst cavity contained keratinized materials. Multinucleated giant cells and macrophages infiltrated in the surrounding cerebellar white matter, in which multilayered keratinization was seen with fibrogliosis.

## Discussion

Previously, there is only one report of intracranial epidermoid cyst in a cat (8). In this report, all results were based on necropsy evaluation. In present case, we performed MRI and surgical treatment and this is the first report for this lesion in a cat. In both cases, the lesions were located adjacent to the fourth ventricle. Similarly, intracranial epidermoid cysts are likely to develop in cerebellopontine angles in humans and dogs (4, 12). While purebred dogs with intracranial epidermoid cysts have been sporadically reported (6, 13), there are no reports suggesting a genetic predisposition. There are currently too few feline cases to comment on breed or genetic predispositions. In dogs, intracranial epidermoid cysts usually occur in adulthood aged 7 years or younger (6, 13, 14). It takes time for these lesions to develop and expand despite a suspected congenital pathology. In humans, they are reported to occur often in middle age as 30–40 years old (3), and in another report they range widely from 16 to 78 years (15). Both feline cases were young to middle aged, consistent with reports in humans and dogs.

In our case, the cyst was depicted on MRI as a well-defined oval cyst beside the fourth ventricle. The inside of the cyst showed high intensity on T2-weighted and FLAIR imaging, low intensity on T1-weighted imaging, and the cyst wall was slightly enhanced by contrast medium in a ring-like pattern. A similar signal pattern has been reported in canine epidermoid cysts, suggesting that such imaging findings may be common in cats and dogs (1, 4). There is very little information when considering the differential diagnosis of cystic lesions in the fourth ventricle in cats because only abscesses and arachnoid diverticula have been reported thus far (10). In dogs, dermoid cysts, choroid plexus cysts and ependymal cysts are other cystic lesions in the fourth ventricle, and can be differentiated from epidermoid cysts by MRI (1, 4, 16, 17). In contrast-enhanced T1W imaging, abscesses show thicker ring-like lesion than epidermoid cysts and choroid plexus cysts show strong and homogeneous contrast enhancement (10, 16). Arachnoid diverticula and ependymal cysts show low intensity on FLAIR imaging (1, 17). Dermoid cysts are similar to epidermoid cysts in that they contain stratified squamous epithelium, but they are distinguished histologically by the presence of organs that make up hair follicles, such as hair, sweat glands, and sebaceous glands in dermoid cysts. That is why dermoid cysts often show heterogeneous high intensity on T1-weighted MRI due to the presence of lipids whereas epidermoid cysts often show low intensity (1, 4). Other mass lesions, not necessarily cystic, that may occur in the fourth ventricle in cats are tumors, including meningioma, lymphoma, ependymoma, and metastatic tumors (11, 18, 19). These tumors often show moderate to fine contrast-enhancement (11, 20) whereas epidermoid cysts usually show slight ring-enhancement. Although definitive diagnosis requires pathological evaluation, MRI findings in our case support a diagnosis of an epidermoid cyst and are comparable to reports in other species.

MRI revealed a second cystic lesion rostral to the cerebellum. This lesion was adjacent to the quadrigeminal cistern and had the same signal pattern as cerebrospinal fluid, consistent with an arachnoid diverticulum. Although few cases in cats with arachnoid diverticulum have been previously reported, they are common in dogs and are incidental findings in more than half cases (21). The cerebellar compression rate in our case was ~30%, which was higher than reported canine cases. However, no significant relationship between the cerebellar compression rate and clinical signs has been demonstrated in dogs (21). Therefore, the clinical contributions of this concurrent lesion compressing the medulla or cerebellum in our case were unknown. Other differentials for this lesion included true cystic lesions such as choroid plexus cysts (22), cystic tumors like meningiomas and replacement by cerebrospinal fluid after cerebellar atrophy. Histopathological examinations are needed to confirm its nature.

In humans, surgical removal of intracranial epidermoid cysts is the treatment of choice, and complete removal of the lesion is expected to result in a good prognosis (2, 15). In the present case, the epidermoid cyst was removed by an occipital approach, but the patient did not regain spontaneous respiration after surgery. This patient was already in respiratory arrest before surgery, suggesting severe medulla injury prior to surgery, but it is unknown if surgery contributed to the lack of improvement or if we simply intervened too late. In dogs, an epidermoid cyst in the fourth ventricle was surgically treated previously, and clinical signs similarly deteriorated after surgery (13). It is difficult to conclude whether surgery is recommended in dogs and cats based on these two reports. In cats, the lateral approach to meningiomas in the cerebellar fossa has been reported, in which no complication was observed (23). This approach is a surgical technique that manipulates the caudal fossa between the tentorium ossium and the nuchal crest from the lateral aspect and is considered to have the advantage of causing less damage to brain parenchyma such as the medulla. This technique may have improved the clinical course of the present case. However, because epidermoid cysts were often located ventral to the cerebellum and covered by cerebellar hemispheres, it may have been difficult to observe the lesions grossly by the lateral approach alone.

In human intracranial epidermoid cysts, incomplete resection of the squamous epithelium composing the cystic wall leads to recurrence, then the prognosis depends on whether complete surgical resection is achieved (2). In dogs, cases of suspected postoperative recurrence have been reported in intracranial and spinal cord epidermoid cysts (13, 24). There are few studies of surgical outcomes and prognosis in animals, and further studies are required to determine the best timing and approach for the surgical treatment of epidermoid cysts.

There are several limitations to the case we reported here. First, the clinical contributions of the rostral cystic-like lesion were still unclear. Any histopathological examination of this lesion was not performed while the excised lesion was diagnosed as an epidermoid cyst. There was a possibility that the cat failed to recover spontaneous respiration due to not only the epidermoid cyst but also the concurrent lesion. Another limitation to our description is the use of a low-field MRI. However, MR imaging in this case are similar to the images in canine case that were also performed with low-field MRI (6). Other reports using high-field MRI showed a slight heterogeneity in the epidermoid cysts (13), which differs in some respects from the images of the present case. Third, the information of the clinical course was limited. The presentation to our department was about 6 weeks after the onset of the disease, and details during this time were lacking. Moreover, we were not able to gain the information on the long-term postoperative course of this case because the patient died after surgery. In order to demonstrate that the surgery may be the standard therapy of intracranial epidermoid cysts in cats, it is necessary to experience a number of successful cases with long-term survival.

In summary, feline intracranial epidermoid cysts are considered histologically benign masses that cause central vestibular disorders due to their anatomic features of predominance around the fourth ventricle. Surgical resection may be necessary for treatment; however, the prognosis is still unclear.

## Data Availability

The original contributions presented in the study are included in the article/supplementary material, further inquiries can be directed to the corresponding author.
